# Diversity analyses of corn leaf aphid *Rhopalosiphum maidis* (Hemiptera: Aphididae) endosymbiotic microbiome and soil microbiome—preliminary results

**DOI:** 10.1093/jisesa/ieag047

**Published:** 2026-06-02

**Authors:** Artúr Botond Csorba, Ciprian G Fora, Adalbert Balog

**Affiliations:** Department of Horticulture, Faculty of Technical and Human Sciences, Sapientia Hungarian University of Transylvania, Târgu Mureș/Corunca, Romania; Faculty of Engineering and Applied Technologies, University of Life Sciences “King Michael I” from Timișoara, Timișoara, Romania; Department of Horticulture, Faculty of Technical and Human Sciences, Sapientia Hungarian University of Transylvania, Târgu Mureș/Corunca, Romania; Institute of Biology, Faculty of Sciences, University of Pécs, Pécs, Hungary

**Keywords:** aphids, adaptation, climate, bacterial symbionts, soil microbiome

## Abstract

The corn leaf aphid, *Rhopalosiphum maidis* Fitch (Hemiptera: Aphididae), microbial symbiont and 9 soil-type microbial diversities were genetically analyzed along a gradient of maize management systems that includes 3 different crop control strategies from 3 climatic regions. The central point of interest was to assess whether any similarity could be detected between the corn leaf aphid’s rapid distribution increase throughout mainland Europe and variation in its endosymbiont microbiome diversity. According to the results, it was detected that the bacterial community differs between regions. The obligate symbiont *Buchnera aphidicola* dominated across all climate regions, while facultative symbionts such as *Serratia symbiotica* and *Wolbachia* varied in relative abundance under different temperature conditions. Fewer effects of soil types were detected. Our study comprises analyses about a pest aphid and its associated symbiont community in relation to ambient temperature conditions, and as such, we believe it may well help in the development of new control strategies.

## Introduction

Insect invasion studies performed under the reality of climate change must consider not only the alien species but also local species. Among these local insects, aphids, especially including agricultural pest expansion under the influence of climate change, have to date not been adequately studied until very recent times (Bonnamour et al. 2023, Zhao et al. 2023). One of the most important up-and-coming local aphid pests in Europe is the corn leaf aphid, *Rhopalosiphum maidis* (Fitch) (Hemiptera: Aphididae) (Blackman and Eastop 2000), its populations having been found to have increased significantly in maize fields during the last 5 years (2019 to 2024) (Csorba et al. 2022). Thus, such a population increase in maize fields and increasing damage and concomitant yield loss have been detected in Germany, Poland, Hungary, and from 2019 onwards, in Romania (Csorba et al. 2022). The corn leaf aphid damage occurs not only as a result of direct physical contact with plants (ie sap-feeding) but also by the transmission of plant pathogenic viruses (Blackman and Eastop 2000, Van Emden and Harrington 2017).

Aphids are closely associated with bacterial symbionts, both obligate and facultative species (Douglas 1998). The obligate species are referred to as primary symbionts, ie *Buchnera aphidicola*, while others are considered facultative or secondary species, the most commonly found being *Serratia symbiotica*, *Wolbachia* spp., *Hamiltonella defensa*, and *Regiella insecticola*. Symbionts are harbored and shared between divergent aphid host lineages by both vertical and horizontal transmission and seemingly may well confer climate and other adaptation mechanisms for host aphid survival (Oliver et al. 2003, Gómez-Valero et al. 2004).

The role of these various symbionts in aphid adaptations, especially in the context of rapid expansions, has not been sufficiently tested until now; moreover, detailed endosymbiont analyses under different climate conditions and soil types representing these climate zones, prevailing during host plant cultivation (maize in our case), have not hitherto been performed. Detailed assessments concerning aphid symbionts along a gradient of climate conditions (excluding our studies of *R. maidis* and its associated endosymbiont arrays) have only been published within the last 20 years or so (Montllor et al. 2002, Tsuchida et al. 2010). Overall, the functional role of these bacterial symbionts in performing a protective role in aphids, a trait that may confer advantages to these insects under specific ecological conditions (eg high and low temperatures), might not only prove to be of fundamental ­scientific importance, but economic importance as well (Douglas 2016).

Until now, the impact of aphid herbivory on the soil microbiome has only been studied to a limited extent. Some studies have reported that aphid herbivory induces alterations in the composition of microbial communities within the rhizosphere; however, other studies found no observable effects between aphid herbivory and host plant soil microbiome diversity, meaning that further research are needed to clarify these discrepancies (O’Brien et al. 2018, Liu et al. 2020). The aphid-dominant microbiome diversity and its host plant microbiome have not been sufficiently assessed until now; however, it has been detected that the composition of the aphid microbiome diversity is influenced by various factors, including the plant host species, geographical location, and the frequency of aphid predators and hymenopterous wasp parasitoids (Zytynska et al. 2021). Some evidence indicates that soil microbial diversity plays a role in shaping the structure of aphid-associated bacterial communities (Zytynska and Weisser 2016), while other studies have identified aphids host plants as a critical factor influencing the composition of the bacterial communities associated with aphids (Xu et al. 2020).

## Objectives

In light of the above factors, the main objectives of the present study and data collection were the following: (i) Detailed assessment of the corn leaf aphids’ endosymbiont diversity under different climate regions; and (ii) assessment of the symbiont variations under different soil types, where maize is cultivated under different climatic conditions. The hypothesis was that soil types under different climate regions and soil microbiomes might have an effect on corn leaf aphid-dominant symbiont diversity. By answering these questions, we could have detailed information about this aphid, and other aphid species’ rapid expansion, and thereby aphid adaptations.

## Materials and Methods

### Study Areas

**Fig. 1.**
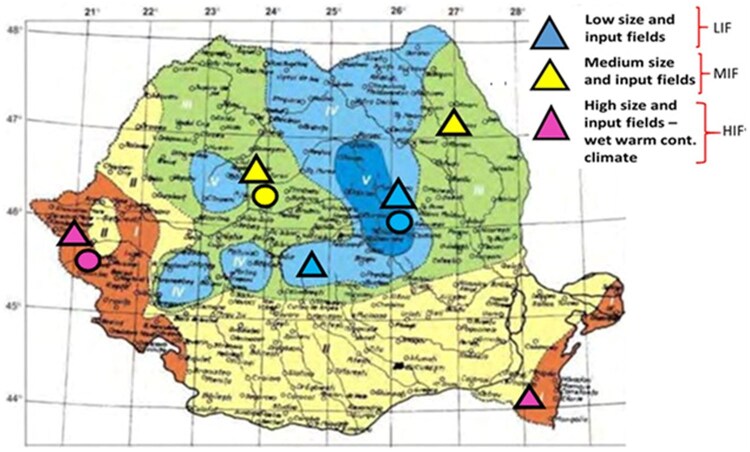
Locations of corn leaf aphid collections in Romania under different maize management systems and climate conditions (triangles), locations of semi-field experiments (dots).

A detailed field assessment and systematic sampling of corn leaf aphids were conducted annually over a 3-year period from 2022 to 2024. The maize fields were selected considering 2 main aspects: the geographic locations and the maize cultivation according to the European maize cultivation systems (Gherghescu and Dabija 2020, Miedaner and Juroszek 2021) (Fig. 1).

In 2022 and 2023, maize fields across southern, southwestern, and eastern Romania (specifically in Timiș and Călărași) (Fig. 1) were sampled, representing regions with warm-wet continental climates (Marcu and Borz 2013), characterized by high-input and large-scale management systems (HIF). These fields were represented as follows: 75,000 grains/ha, seed treatment included fungicide and Nuprid 10 l/t, fertilization at seedbed preparation urea 250 kg/ha, fertilization at sowing 14:28:14 300 kg/ha, herbicide Adengo 0.4 l/ha pre-emergent, herbicide Nicogan 1.5 l/ha + Concordia 0.5 l/ha post-emergent.

Fields in the eastern and mid-western parts of Romania (including lași and Cluj) were selected to represent temperate continental climates (Marcu and Borz 2013) and medium-size, medium-input maize management systems (MIF).

The average interventions and field characteristics were as follows: 68,000 grains/ha, seed treatment—Prothioconazole + Metalaxyl, herbicide pre-emergent: Isoxaflutole + Thiencarbazone-methyl, fertilizers—50 t/ha organic fertilizer at seeding, and at the 12 leaf stage—inorganic fertilizer, NH4NO3 + CaMg (CO_3_) 2,200 kg/ha.

Additionally, fields from central Romania (Brașov and Covasna) were sampled, exemplifying cool continental climate regions (Marcu and Borz 2013) located in mountainous areas and characterized by low-input and small-scale maize management system (LIF) (Fig. 1). These fields were represented by: 68,000 grains/ha, fertilizers—50 t/ha organic fertilizer at seeding.

Altogether, these regions represented the whole climate and management systems of maize cultivation in Europe, and probably 80% of all maize cultivation regions from the globe’s northern hemisphere.

The abbreviations used for sites, as well as management systems and soil types, are presented in Fig. 1 and Table 1.

**Table 1.** Presentation of sampling data for areas of different maize management systems and climate conditions, respectively, and soil types in each climate zone

Locations	Treatment	Samples collected	Samples analyzed genetic	Genetic reads used for data analyses
**Timiș (HIF)**	High size and input fields (HIF)—warm continental climate (summer between 20 and 38 °C).	120	60	<6 million reads
**Călărași (HIF)**	High size and input fields (HIF)—warm continental climate.	60	60	<6 million reads
**Cluj (MIF)**	Medium size and input fields (MIF)—temperate continental climate (summer between 18 and 30 °C).	60	60	<6 million reads
**Iași (MIF)**	Medium size and input fields (MIF)—temperate continental climate	60	60	<6 million reads
**Brașov (LIF)**	Low size and input fields (LIF)—cool continental climate (summer between 16 and 22 °C).	120	60	<4 million reads
**Covasna (LIF)**	Low size and input fields (LIF)—cool continental climate	120	60	<4 million reads
**Soil samples HIF**	3 soil type and 1 control	144	144	<6 million reads
**Soil samples MIF**	3 soil type and 1 control	144	144	<6 million reads
**Soil samples LIF**	3 soil type and 1 control	144	144	<6 million reads

In 2024, a semi-field experiment was established in the 3 previously presented geographic regions, representing 3 climate areas. During this experiment, 96 maize plants per site were potted and kept under field conditions. Under each climate region, from 96 plants, 4 blocks were created (of 24 plants each), and 4 soil types were used as substrate for maize, 3 of these representing the characteristic soil for the region and 1 garden soil (same in all regions) as a control. The names and abbreviations are as follows: T-CZ—Cernoziom cambic (CZcb-RST) Cambic Chernozem (WRB), T-PS—Preluvosol stagnic (ELst-RST) Stagnic luvisol (WRB), and T-AS—Aluviosol gleic (ALgc-RST) Gleyi fluvisols (WRB) from warm-wet continental climate, M-EL—Preluvosol Stagnic (RST) Stagnic Preluvosol (WRB), M-GS—Gleisol (Gs-RST) Gleysol (Dystric Gleysol) (WRB), and M-AS—Aluviosol entic (ASen—RST) Fluvisol (WRB) from temperate continental climate, S-FZ—Faeoziom (Fz-RST) Faeozem (WRB), S-AS—Aluviosol coluvic (As co-RST) Colluvic Fluvisol (WRB), and S-RS—Regosol calcaric (Rs ca) Calcaric Regosol (WRB) from cool continental climate (Fig. 1). From all 24 sets of plants cultivated on the same soil type, 4 semi-blocks of 6 maize plants each were randomly separated, representing 4 replicates per 1 soil type per region (Fig. 1). The same sweet maize hybrid (Dessert R68, F1) was used in each region and soil type, and after germination, all pots were connected to the automatic irrigation system, with the capacity of 2 liters of water/h in all regions, and managed during the whole experimental time period. Also, the fertilization method used under normal management conditions was applied, and NPK fertilizer (Plantafol 30-10-10) was used twice during the vegetation period. The first application was made on 15 May, and the second on 10 June with the same dosage (200 g/100 liters of water).

### Field Collection of Corn Leaf Aphids

Aphid samples were collected between the same time period every year (June and July) when the maize plots were in flowering stages (R2 to R3). The BBCH scale classification and phenological phase of the host plants at the time of sampling were characterized as follows: Flowering (6)—Beginning of pollen shedding, appearance of stamens (63) and Full flowering (50%)—lower and upper parts of the crest are in bloom, stamens are fully emerged (65) (Lancashire et al. 1991). All samples were taken from maize plants within the interior of the fields to minimize potential edge effects.

In 2022 to 2023, corn leaf aphids were collected from 6 maize fields, with each field sampled in 2 replicates, and within each field, 2 smaller plots (sites) were selected and sampled to ensure representative data collection. Consistent sampling procedures and methods were applied across all sites, and asexual lineages of wingless (apterous) individuals were chosen for collection. At 3 sampling dates, 10 maize plants per field were randomly selected. From each selected plant, first instar aphid nymphs (5 from each colony per plant) were collected and stored in 0.5 ml Eppendorf tubes containing 99% ethanol, in preparation for subsequent DNA analysis. This was done because of telescopic generations in aphids; first instars do not have embryos developed in their bodies, and because we were interested in assessing symbionts that are vertically transmitted, the methodology of testing first instars was the best way to not bias our results.

Under a semi-field experiment in 2024, during the vegetative growing period, before aphid colonization, all plants were checked, and any possible insect damage was assessed. During the maize flowering time (in May under warm climate and in June under cold climate), all plants were colonized with corn leaf aphids by infected plants from the fields representing the regions where the previous year’s sampling was made. Infected maize plants selected were cut and removed to the designated experimental field. Plant fragments were then placed near the experimental maize plants to allow apterous aphid individuals to move onto them, either by walking or being carried by ants. A total of 3 sampling periods were conducted, during which apterous first-instar larvae were collected for subsequent symbiont genetic analyses. The first collection time was made after each plant had formed well-established and reproducing aphid colonies. The second collection was conducted 2 weeks later, followed by a third and final sampling after another 2 weeks, ensuring that aphids from distinct generations were obtained at each sampling event. From each set of 6 plants/soil types, 2 plants from the same 2 replicates (4 as total) were selected randomly, and aphids were collected (ie 16 samples per 1 region per 1 collection data per soil type). In this way, a total of 144 aphid samples for symbiont genetic analysis were sampled and genetically analyzed (Table 1). At each aphid sampling, soil samples were also collected, and from each soil type, including garden soil, 2 samples per period (from the same pot with maize plant) were collected in ethanol and kept in −70 °C until analyses. Aphid samples were also collected in ethanol and kept in −70 °C until analysis.

### Aphid-Associated Bacterial Community Analysis

A detailed analyses of the endobacterial symbionts of *R. maidis* have been described in our previously published paper (Csorba et al. 2022).

#### 2022 to 2023

The methods presented here refer to the processing methods for samples collected in 2022 and 2023. Partial sequences of the 16S rRNA gene were amplified from aphid DNA extracts using specific primer pairs (Bakt_341F [5′-CCT ACG GGN GGC WGC AG-3′] [Herlemann et al. 2011] and Bakt_805NR [5′-GAC TAC NVG GGT ATC TAA TCC-3′] [Apprill et al. 2015]), designed for the hypervariable regions of the gene (V2-4-8 and V3-6, 7-9). PCR products targeting these specific regions of the 16S rRNA gene were purified using Agencourt AMPure beads. Subsequently, libraries were constructed using the Ion Plus Fragment Library kit (Applied Biosystems), and their concentrations were quantified using the Ion Universal Library Quantitation kit (Cat no. A26217). Template preparation was completed using the ION PGM Hi-Q View OT2 kit-400, followed by sequencing of the amplicon libraries on a 318-chip using the Ion Torrent PGM system. Sequence reads obtained were initially processed using Ion Reporter PGM software to remove polyclonal and low-quality reads. Subsequent analysis of the sequencing data was conducted using Quantitative Insights Into Microbial Ecology (Bolyen et al. 2018). Operational Taxonomic Units (OTUs) of the 16S rRNA gene were defined at a threshold of ≥97% sequence homology to calculate downstream diversity measures. Taxonomic classification of all reads was performed using curated reference datasets (Curated Greengenes v13.5; Curated MicroSEQ 16S Reference Library v2013.1).

#### 2024

Aphids and soil samples from semi-field experiment from 2024 were analyzed using Illumina PE reads trimmed with cutadapt V3.5 (Martin 2011). Amplified sequences of the 16S rRNA gene from first instar aphid DNA extracts were completed using primer pairs designed for the hypervariable regions of the V2-4-8 and V3-6, 7-9 genes. The resulting PCR products of the specific regions of 16S rRNA gene were purified with Agencourt AMPure beads (Beckman Coulter). Libraries were constructed using the Ion Plus Fragment Library kit (Applied Biosystems), and their concentration was determined using the Ion Universal Library Quantitation kit (Cat no. A26217). Untrimmed reads were not considered in the further analysis. Sequence level error estimation, filtering, merging, and taxonomy assignment were completed with DADA2 workflow V1.22 (Callahan et al. 2016). Silva 16S NR99 V138.2 database was used to assign taxa on the species level (Quast et al. 2013). Sample ID abbreviations and tracking reads are given in the Supplementary Tables S1 to S6 .

### Data Analyses

2022 to 2023 (Ion Torrent) and 2024 (Illumina) datasets were analyzed separately. Comparisons across years were qualitative not quantitative. The methodological details and the bioinformatic and statistical analyses applied were performed as described in our previously published articles (Csorba et al. 2022, 2025) and in Benedek et al. (2019), except that the resulting sequence reads were processed using mothur v1.41 software (Schloss 2020). This was based on the MiSeq standard operating procedure, with the removal of chimeric sequences performed using VSEARCH (Rognes et al. 2016). OTUs were defined at a 97% nucleotide sequence similarity level. Raw sequence data were submitted to NCBI under BioProject ID PRJNA647165. Statistical analysis of the amplicon sequencing data included subsampling reads to match the read number of the smallest dataset (*n* = 56,288). Microbial alpha diversity indices of both aphids and soil microbiomes and microbial species richness values were assessed utilizing the Chao1 and ACE richness metrics calculated with mothur v1.41. Endosymbiotic taxa were ranked based on their abundance (total genomic DNA) by the differences observed between management systems. Data are presented for taxa contributing >0.1% to the overall composition. Because no differences in dominant microbiome community inside soil types per region were detected, these data are presented as pooled data. Comparisons across years are not quantitatively robust because the datasets were generated using different sequencing platforms, so any apparent temporal patterns are presented as qualitative data.

Principal Coordinates Analysis (PCoA) was used to test the effect of climate conditions (sites) and soil types on bacterial symbiont abundances, where sites and soil types were considered as main components and endosymbiont species abundances as variables.

To quantify the differences in microbial community composition between regions and soil types, beta diversity indexes as Jaccard and Bray–Cutis dissimilarities were computed for both aphids’ symbionts and soil microbiomes. Because of the high differences between samples, only aphids’ symbionts tested under field conditions and Bray–Curtis for aphids’ symbionts under semi-field experiments were used. The analyses were made in Past 4.1.

All the collected areas presented in Table 1 are separately uploaded, and datasets of the corn leaf aphid microbiome are presented in OUT at species, genus, and family levels in Figshare. Genetic reads (sequences of each bacterial taxa) in fasta file at family, genus, and species levels are given for each location. Table 1 summarizes the number of corn leaf aphid samples collected during 2022 to 2023, along with those obtained from the semi-field experiment in 2024, as well as the subset of samples subjected to genetic analysis for microbiome assessment and the corresponding number of sequencing reads used in downstream analyses. A PERMANOVA analysis of the semi-field experiments sample diversities between generations (D1, D2, D3) and soil types was computed for each region in the R V4.1.2 environment. Results are presented in Supplementary Tables S7 to S33.

## Results

### Corn Leaf Aphid Endosymbiont Diversity

**Fig. 2.**
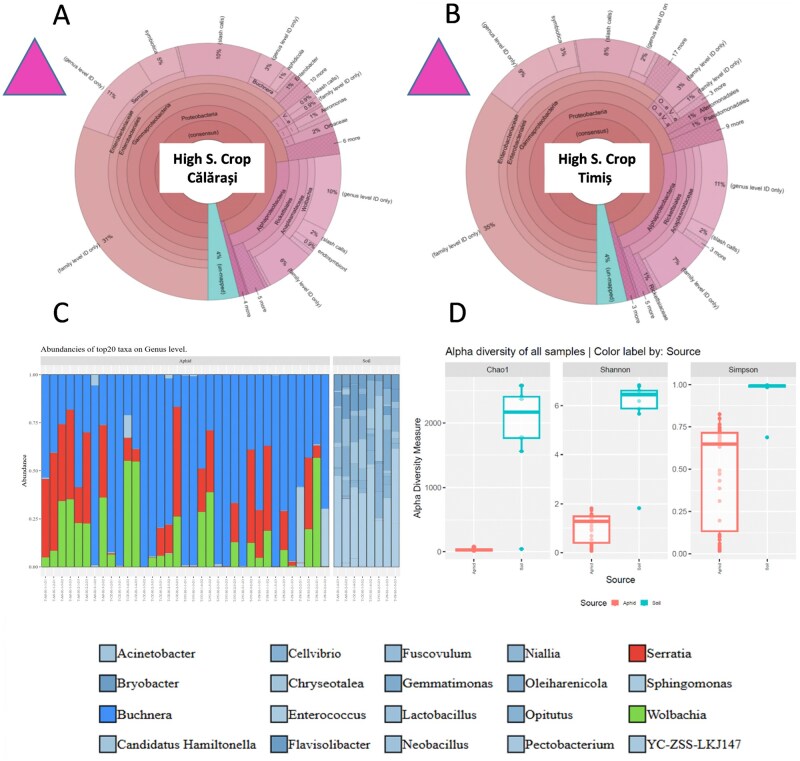
Corn leaf aphid microbial symbiont and its host plant soil microbiome abundances under open-field (A and B) and semi-field experimental conditions (C), and alpha diversity indices of aphids and soil microbiomes (D) under warm climate conditions and high input management maize crops. For open-field data, Operational Taxonomic Units (OTUs) of the 16S rRNA gene were defined at a threshold of ≥97% sequence homology to calculate downstream diversity measures. Taxonomic classification of all reads was performed using curated reference datasets (Curated Greengenes v13.5; Curated MicroSEQ 16S Reference Library v2013.1). For semi-field data, Silva 16S NR99 V138.2 database was used to assign taxa on species level. Microbial alpha diversity indices of both aphids and soil microbiomes and microbial species richness values were assessed utilizing the Chao1 and ACE richness metrics calculated with mothur v1.41. Comparisons across years are not quantitatively robust because the datasets were generated using different sequencing platforms, so any apparent temporal patterns are presented as qualitative data. Direct numerical comparisons between the 2022, 2023, and 2024 panels should not be made.

Due to differences in sequencing platforms, primer sets, and bioinformatic pipelines, cross-year comparisons between symbionts can be interpreted cautiously and limited to broad compositional patterns rather than direct quantitative comparisons. For each set of locations grouped by similar climate conditions (Tables 2 to 4), the composition of the most frequent bacterial symbionts is shown. This includes DNA read frequencies (field data from 2022 and 2023) and the corresponding percentages for each symbiont, calculated from read frequencies per sample, across the different climate conditions and soil types. Only the 10 most frequent taxa at the species level are presented. The 10 most frequent families represented approximately 90% of all families identified in high-size plots under a warm, wet continental climate (HIF). The primary symbiont *B. aphidicola* dominated, followed by the secondary symbiont *S. symbiotica* and *Wolbachia* spp (Fig. 2A and B;  Table 2).

**Table 2.** Corn leaf aphid bacterial symbiont frequency (their abundance in total genomic DNA) under field conditions in Calarasi and Timis under high-input field and wet warm continental climate (HIF) according to the genetic reads (sequences of each bacterial taxa), and symbionts percentages under different soil type where maize is cultivated under this climate zone and control soil (Silva 16S NR99 V138.2 database)

Symbiont species	Calarasi	Timis	T-AS (Aluviosol)	T-PS (Preluvosol)	T-CZ (Cernoziom)	T-PG (Control garden soil)
***Serratia symbiotica***	51	81	35%	31%	34%	36%
***Wolbachia endosymbiont***	16	24	29%	24%	26%	24%
***Buchnera aphidicola***	12	9	17%	19%	21%	23%
***Enterobacter cloacae***	8	10	3%	2%	3%	1%
***Klebsiella singaporensis***	2	0	1%	1%	1%	1%
***Serratia marcescens***	2	3	1%	1%	1%	1%
***Vibrio neocaledonicus***	2	1	1%	1%	1%	1%
***Halomonas* sp.**	1	0	1%	1%	1%	1%
***Citrobacter freundii***	1	0	1%	1%	1%	1%
***Klebsiella pneumoniae***	1	4	1%	1%	1%	1%

The most frequent 10 species are presented.

### Symbiont Variation Under Different Climate and Soil Types

The same trend in symbiont dominance was detected when symbiont frequencies were compared by soil types representing the warm regions (Fig. 2C). No clear similarity was observed between aphid and soil microbiomes in this climatic region; instead, soil communities were dominated at the genus level and exhibited distinct alpha diversity patterns compared to those associated with aphids (Fig. 2C and D).

**Fig. 3.**
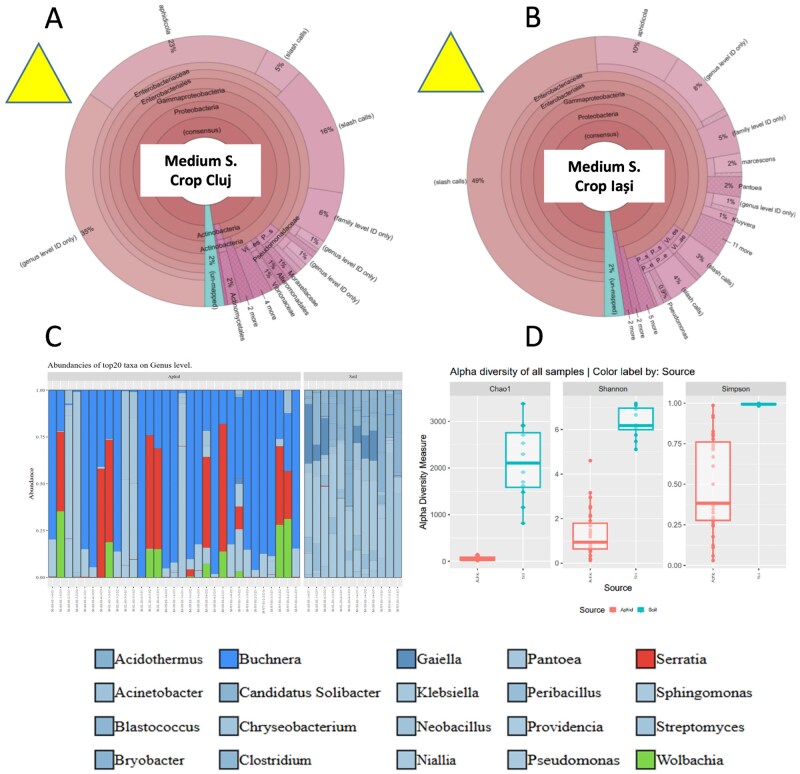
Corn leaf aphid microbial symbiont and its host plant soil microbiome abundances under open-field (A and B) and semi-field experimental conditions (C), and alpha diversity indices of aphids and soil microbiomes (D) under temperate climate conditions and medium input management maize crops. For open field data, Operational Taxonomic Units (OTUs) of the 16S rRNA gene were defined at a threshold of ≥97% sequence homology to calculate downstream diversity measures. Taxonomic classification of all reads was performed using curated reference datasets (Curated Greengenes v13.5; Curated MicroSEQ 16S Reference Library v2013.1). For semi-field data, Silva 16S NR99 V138.2 database was used to assign taxa on species level. Microbial alpha diversity indices of both aphids and soil microbiomes and microbial species richness values were assessed utilizing the Chao1 and ACE richness metrics calculated with mothur v1.41. Comparisons across years are not quantitatively robust because the datasets were generated using different sequencing platforms, so any apparent temporal patterns are presented as qualitative data. Direct numerical comparisons between the 2022, 2023, and 2024 panels should not be made.

Medium size and input fields at temperate continental climate (MIF) at species level were clearly dominated by the *B. aphidicola*, and the secondary symbionts *S. symbiotica* and *Wolbachia* were present only in much lower frequency; again, the same trend under different soil type in this region was detected (Fig. 3A and B;  Table 3). Soil microbiomes were dominated by *Niallia*, *Sphingomonas*, *Pantoea*, and *Peribacillus*, and again, no clear similarity between soil and aphid microbiomes was detected (Fig. 3C and D).

**Table 3.** Corn leaf aphid bacterial symbiont frequency (their abundance in total genomic DNA) under field conditions in Cluj and Iasi under medium size and input fields at temperate continental climate (MIF) according to the genetic reads (sequences of each bacterial taxa), and symbionts percentages under different soil type where maize is cultivated under this climate zone and control soil (Silva 16S NR99 V138.2 database)

Symbiont species	Cluj	Iasi	M-AS (Aluviosol entic)	M-EL (Preluvosol argic stagnic)	M-GS (Gleyosol)	M-VF (Control garden soil)
***Buchnera aphidicola***	60	64	78%	77%	75%	75%
***Serratia symbiotica***	19	13	9%	9%	8%	8%
***Wolbachia endosymbiont***	11	9	8%	7%	6%	6%
***Microbacterium oxydans***	1	0	1%	1%	1%	1%
***Pseudomonas frederiksbergensis***	1	0	1%	1%	1%	1%
***Allomonas enterica***	0	1	1%	1%	1%	1%
***Buttiauxella* sp.**	0	1	1%	1%	1%	1%
***Citrobacter gillenii***	0	1	1%	1%	1%	1%
***Cronobacter sakazakii***	0	2	1%	1%	1%	1%
***Serratia marcescens***	0	14	1%	1%	1%	1%

The most frequent 10 species are presented.

**Fig. 4.**
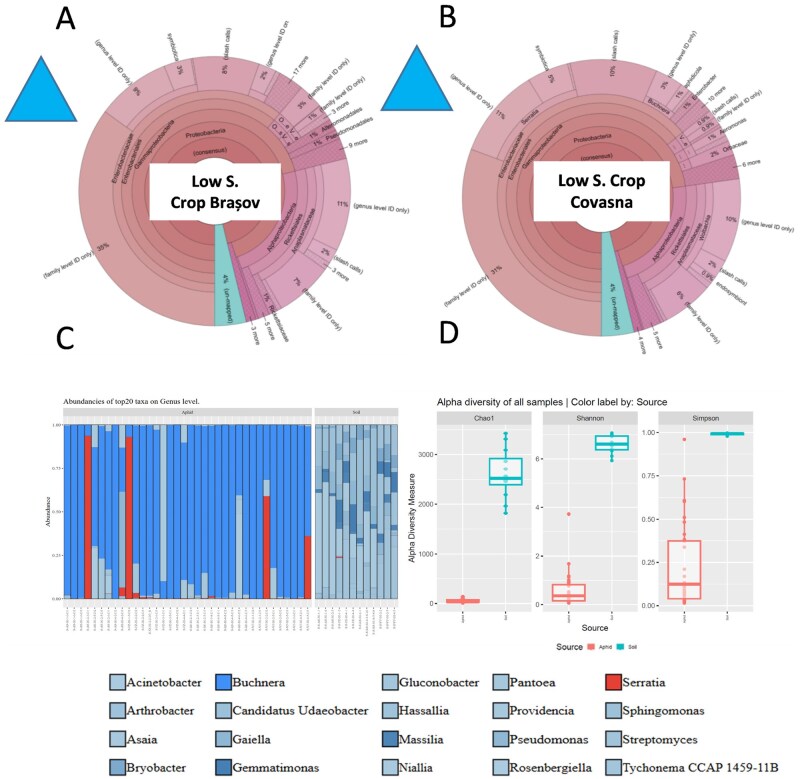
Corn leaf aphid microbial symbiont and its host plant soil microbiome abundances under open-field (A and B) and semi-field experimental conditions (C), and alpha diversity indices of aphids and soil microbiomes (D) under cool climate conditions and low input management maize crops. For open field data, Operational Taxonomic Units (OTUs) of the 16S rRNA gene were defined at a threshold of ≥97% sequence homology to calculate downstream diversity measures. Taxonomic classification of all reads was performed using curated reference datasets (Curated Greengenes v13.5; Curated MicroSEQ 16S Reference Library v2013.1). For semi-field data, Silva 16S NR99 V138.2 database was used to assign taxa on species level. Microbial alpha diversity indices of both aphids and soil microbiomes and microbial species richness values were assessed utilizing the Chao1 and ACE richness metrics calculated with mothur v1.41. Comparisons across years are not quantitatively robust because the datasets were generated using different sequencing platforms, so any apparent temporal patterns are presented as qualitative data. Direct numerical comparisons between the 2022, 2023, and 2024 panels should not be made.

Corn leaf aphid microbial symbionts reared under a cool continental climate, and low size input field (LIF) conditions were in some content similar to those from HIF (the 2 extreme climate conditions in terms of corn leaf aphid distributions), and high dominance of *B. aphidicola* and *S. symbiotica* was observed; *S. marcescens* was present at lower frequency, and *Wolbachia* spp. missed completely from this region. Again, the same trend under different soil types were detected (Fig. 4A and B; Table 4). Soil microbiomes were dominated by *Niallia*, *Pantoea*, and *Candidatus*, and again low similarity between soil and aphids microbiomes was detected (Fig. 4C and D).

**Table 4.** Corn leaf aphid bacterial symbiont frequency (their abundance in total genomic DNA) under field conditions in Brasov and Covasna under cool continental climate and low size input field (LIF) according to the genetic reads (sequences of each bacterial taxa), and symbionts percentages under different soil type where maize is cultivated under this climate zone and control soil (Silva 16S NR99 V138.2 database)

Symbiont species	Brasov	Covasna	S-AS (Aluviosol coluvic)	S-FZ (Faeoziom)	S-RS (Regosol calcaric)	S-VF (Control garden soil)
***Serratia symbiotica***	14,323	20,655	47%	43%	42%	45%
***Serratia marcescens***	11,232	1,750	9%	6%	7%	5%
***Wolbachia endosymbiont***	0	0	0%	0%	0%	0%
***Microbacterium saperdae***	120	146	1%	1%	1%	1%
***Buchnera aphidicola***	99	109	22%	24%	24%	25%
***Sphingomonas hankookensis***	34	42	1%	1%	1%	1%
***Sphingomonas panni***	22	33	1%	1%	1%	1%
***Sphingomonas yabuuchiae***	23	22	1%	1%	1%	1%
***Serratia ureilytica***	12	17	1%	1%	1%	1%
***Sphingomonas cynarae***	11	16	1%	1%	1%	1%

The most frequent 10 species are presented.

**Fig. 5.**
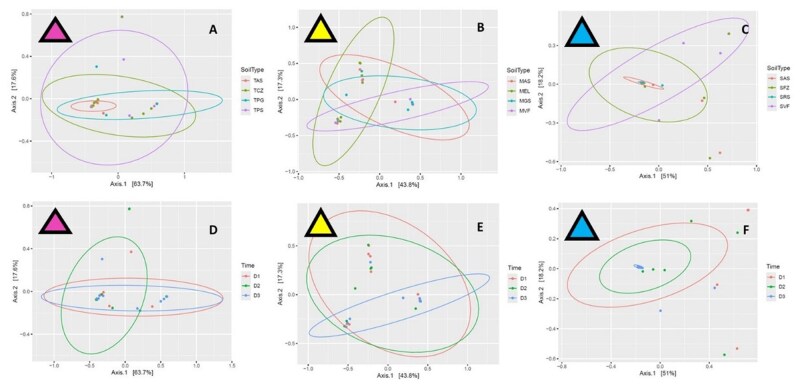
Principal Coordinates Analyses (PCoA) comparing climate conditions (sites) and soil types on bacterial symbionts abundance by soil types (A to C) and generations (D to F) under different climate conditions.

Using PCoA to test the effect of climate conditions (sites) and soil type on bacterial symbiont abundance, it was found that the site (climate effects) determined symbiont abundance, being >63.7% in warm regions, >43.8% in temperate regions, and >51% in cold regions, while soil type had only around 17% to 18% effect (Fig. 5A to C). The same trends were detected upon testing these variations between generations, where again the site effects mainly determined the symbiont abundance, whilst soil type had a lower effect (Fig. 5D to F).

**Fig. 6.**
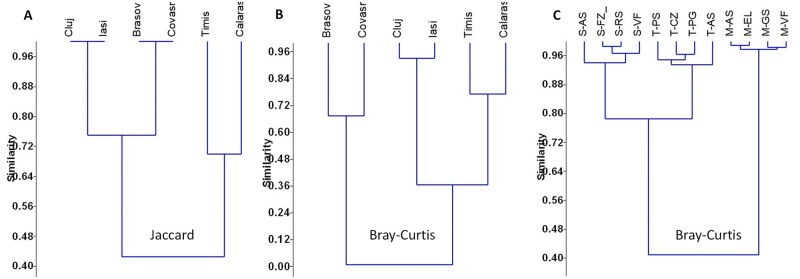
Beta diversity indices (Jaccard) (A) and Bray–Curtis (B) of the aphids’ symbionts under open field conditions, and Bray–Curtis dissimilarity (C) under semi-field experiment. Beta diversity dissimilarity indexes were computed using Past 4.1. Indexes ranges from 0 to 1, where 0 indicates identical composition and 1 indicates complete dissimilarity composition of microbiomes.

Beta diversity also revealed similarity in aphid symbionts between climatic regions (both Jaccard and Bray–Curtis under open field conditions) and also between soil types; here only Bray–Curtis were possible to be computed (Fig. 6A to C). PERMANOVA analyses also revealed no differences in symbiont diversity between generations and soil types (see Supplementary Tables S7 to S33).

## Discussion

Our results provide preliminary insights into variation in aphid-associated symbiont communities across environmental gradients. Because aphids used in each climatic region were collected locally and thus carried distinct initial symbiont communities, the observed regional differences in the semi-field experiment may reflect source effects in addition to climate. Causal attribution is therefore not possible. Further experimental studies are required to determine functional roles and potential applications in pest management. The corn leaf aphid and soil microbiome assessment and the functional groups of obligate and facultative microbial symbionts species composition under different climate conditions and management systems were analyzed by comparing microbial diversity of both symbionts and at the soil level. It is important to underline that because climate, geography, management intensity, and soil type co-vary in this study design, the observed patterns should be interpreted as associations rather than causal effects of climate.

Similar works made at a smaller spatial scale also noted that the primary symbiont, *Buchnera*, as well as a few facultative species (*Serratia*, *Wolbachia*) dominated corn leaf aphid communities (Csorba et al. 2022). Some previous studies reported that aphid symbiont diversity might also be influenced by soil type; however, no clear evidence of these results was demonstrated in this study (Qin et al. 2021). Not even when control soil types are compared, its microbiome diversity is rather similar to the other soils’ microbiomes in the same climate regions.

No differences were detected between endosymbiont diversity inside regions. *Serratia symbiotica* but also *Wolbachia* were demonstrated to be involved in defense against heat and potentially in aphid nutrition (Xu et al. 2021). An earlier study, assessing the endosymbiotic bacterial diversity of the sugarcane aphid, *Melanaphis sacchari* (Zehntner) and podocarpus aphid, *Neophyllaphis podocarpi* (Takahashi), revealed that altitude was negatively associated with *S. symbiotica* richness; therefore, it was concluded that *S. symbiotica* protected aphids from heat stress (in both directions) by regulating the aphid (Xu et al. 2021). This can be the explanation why *S. symbiotica* was present in all climate regions.

In the present study, the abundance of *Wolbachia* decreased under a continental climate and was completely missed under cool conditions. Other studies also demonstrated that cool temperature reduces *Wolbachia* oocyte abundance and maternal transmission (Hague et al. 2022).

The presence of Wolbachia was first reported in the conifer aphid, *Cinara cedri* Mimeur, by Gómez-Valero et al. (2004), and some protective role has been attributed, including protection against some pathogens and also against extreme heat stress in other insect systems (Iturbe-Ormaetxe et al. 2011).

Several other factors may have influence on aphids’ symbiont-specific compositions and can alter soil microbiomes. In the present study, the soil samples were pooled, which limits inference about soil type effects. Studies on insecticide application in the pea aphid (*Acyrthosiphon pisum* Harris) have demonstrated that the secondary symbiont *S. symbiotica* may contribute to the degradation and/or detoxification of insecticides, including imidacloprid, chlorpyrifos-methyl, methomyl, cyantraniliprole, and spirotetramat. Therefore, it was hypothesized that *S. symbiotica* may promote resistance to chemicals; however, it was detected that *Serratia*-infected aphids were more susceptible to most of the tested insecticides than non-infected aphids (Skaljac et al. 2018). In light of these findings, evidence exists that the endosymbiont bacterial diversity of corn leaf aphid is related to, and influenced by, climate conditions and probably is less dependent on soil type and host aphid generation, as revealed by PCoA.

Another factor such as the landscape structure may also have a positive or negative impact on insect (aphids) symbionts’ diversity (Zytynska and Meyer 2019). A complex assessment using a large-scale field trial, which assessed the bacterial endosymbiont communities of the cereal aphids *Sitobion avenae* revealed that floral plantings and flower identity had significant effects on the species compositions of bacterial endosymbiont, and it was also correlated with natural enemy diversity and abundance (Zytynska et al. 2023).

## Conclusions

In light of these findings, we can conclude that endosymbiont bacterial diversity of corn leaf aphid is much more related to several complex factors. Because climate, geography, management intensity, and soil type co-vary in this study design, the observed patterns should be interpreted as associations rather than causal effects of climate.

Our results are important not only in terms of the fundamental biology of this aphid species in relation to its adaptation to different environmental-ecological conditions.

From a practical and scientific point of view, because aphid data have been collected under different climate and management systems in maize production, more detailed analyses of the role of facultative symbionts can be made and analyzed in the future as follows:

- New studies of corn leaf aphid bacterial microbiome distributions along a much higher gradient and climate spectrum can be made.
- Comparison of corn leaf aphid microbiomes with other aphid species microbiomes, with importance in crop management can be computed.
- New field methods can be implemented in terms of aphid pest control by focusing on those bacterial symbionts that confer real adaptations in terms of aphid distributions along a gradient of climate conditions.

## Supplementary Material

ieag047_Supplementary_Data

## Data Availability

The datasets used and/or analysed during the current study are available at: https://mega.nz/folder/XVUnyKSI#3ABxJ5s3SdEzMWFRtgWSnA
