# Supplementary material for: Diversity analyses of corn leaf aphid Rhopalosiphum maidis (Hemiptera: Aphididae) endosymbiotic microbiome and soil microbiome—preliminary results
Source: J Insect Sci. 2026 Jun 2;26(3):ieag047. doi: 10.1093/jisesa/ieag047 (PMC13228132; doi:10.1093/jisesa/ieag047)
Supplement: ieag047_Supplementary_Data [file ieag047_supplementary_data.docx]

**Supplementary online materials**

**Diversity analyses of corn leaf aphid *Rhopalosiphum maidis Fitch* (Hemiptera: *Aphididae*) endosymbiotic microbiome and soil microbiome – preliminary results**

**Abbreviations used for 2024 data in Figshare – Illumina**

**Table S1. Sample IDs from semi-field experiments under high input files and warm climate conditions**

**SampleID Name Source**

3622-T-PS-BI-1-1-D1 T-PS-BI-1-1-D1 Aphid

3623-T-AS-BI-1-2-D1 T-AS-BI-1-2-D1 Aphid

3626-T-CZ-BI-1-2-D1 T-CZ-BI-1-2-D1 Aphid

3630-T-PG-BI-1-1-D1 T-PG-BI-1-1-D1 Aphid

Where the first 4item number represents the order in analyses, T – location code, here Timis, PS – preluvosol, AS – aluviosol, CZ – cernoziom, PG – control garden soil, Bl-1-1 means block 1, plant number 1, from that soil type, D1 means fist sampling period.

**Table S2. Tracking reads through the workflow from the data from warm climate.**

**Sample FWD_in FWD_ctdpt REV_in REV_ctdpt denoisedF denoisedR merged nonchim**

3622-T-PS-BI-1-1-D1 126058 116722 126058 116722 109324 109445 107827 103081

3623-T-AS-BI-1-2-D1 86938 82850 86938 82850 77510 77511 76973 76620

3626-T-CZ-BI-1-2-D1 131861 124997 131861 124997 117309 117557 116309 114245

3630-T-PG-BI-1-1-D1 127052 112087 127052 112087 104801 104618 103376 102514

Where

FWD_in: total number of the forward reads used in the analysis.

FWD_ctdpt: Total number of the forward reads passed the cutadapt primer trimming process

REV_in: total number of the reverse reads used in the analysis.

REV_ctdpt: Total number of the reverse reads passed the cutadapt primer trimming process.

denoisedF: Total number of the forward reads passed the DADA2 trimming and filtering process

denoisedR: Total number of the reverse reads passed the DADA2 trimming and filtering process

merged: : Total number of the successfully merged reads

nonchim: Total number of the merged and nonchimeric reads

During the DADA2 workflow 15855 unique amplicon sequence variants (ASV) were found in 43 samples. Out of

the 15855 ASVs the process idetified 4187 chimera sequences and 686 ASVs that were shorter than the minimum

length (390 nt) or longer than the maximum length (500 nt).

…………………………………………………………………

**Table S3. Sample IDs from semi-field experiments under medium input files and temperate climate conditions**

**SampleID Name Source**

3658-M-EL-BI-4-3-D1 M-EL-BI-4-3-D1 Aphid

3659-M-GS-BI-4-3-D1 M-GS-BI-4-3-D1 Aphid

3666-M-AS-BI-1-4-D1 M-AS-BI-1-4-D1 Aphid

3660-M-VF-BI-2-2-D1 M-VF-BI-2-2-D1 Aphid

Where the first 4item number represents the order in analyses, M – location code, here Mures (Cluj region), EL – preluvosol argic stagnic, GS – gleisoil, AS – aluviosol entic, VF – control garden soil, Bl-4-3 means block 4, plant number 3 from that soil type, D1 means fist sampling period.

**Table S4. Tracking reads through the workflow from the data from temperate climate.**

**Sample FWD_in FWD_ctdpt REV_in REV_ctdpt denoisedF denoisedR merged nonchim**

3658-M-EL-BI-4-3-D1 139541 120045 139541 120045 109516 110071 107492 104107

3659-M-GS-BI-4-3-D1 145356 124132 145356 124132 107703 108802 101818 97207

3666-M-AS-BI-1-4-D1 128899 113417 128899 113417 104452 104341 102295 101355

3660-M-VF-BI-2-2-D1 115105 101120 115105 101120 92232 92122 91179 90001

Where

FWD_in: total number of the forward reads used in the analysis.

FWD_ctdpt: Total number of the forward reads passed the cutadapt primer trimming process

REV_in: total number of the reverse reads used in the analysis.

REV_ctdpt: Total number of the reverse reads passed the cutadapt primer trimming process.

denoisedF: Total number of the forward reads passed the DADA2 trimming and filtering process

denoisedR: Total number of the reverse reads passed the DADA2 trimming and filtering process

merged: : Total number of the successfully merged reads

nonchim: Total number of the merged and nonchimeric reads

During the DADA2 workflow 26217 unique amplicon sequence variants (ASV) were found in 43 samples. Out of

the 26217 ASVs the process idetified 6905 chimera sequences and 2807 ASVs that were shorter than the minimum

length (390 nt) or longer than the maximum length (500 nt).

…………………………………………………………………

**Table S5 Sample IDs from semi-field experiments under low input files and cool climate conditions**

**SampleID Name Source**

3587-S-AS-BI-2-6-D1 S-AS-BI-2-6-D1 Aphid

3593-S-RS-BI-4-5-D1 S-RS-BI-4-5-D1 Aphid

3594-S-FZ-BI-4-4-D1 S-FZ-BI-4-4-D1 Aphid

3586-S-VF-BI-2-5-D1 S-VF-BI-2-5-D1 Aphid

Where the first 4item number represents the order in analyses, M – location code, here Sepsi (Covasna region), AS – aluviosol coluvic, RS – regosol calcaric, FZ – gfaeosiom, VF – control garden soil, Bl-2-6 means block 2, plant number 6 from that soil type, D1 means fist sampling period.

**Table S6. Tracking reads through the workflow from the data from cool climate.**

**Sample FWD_in FWD_ctdpt REV_in REV_ctdpt denoisedF denoisedR merged nonchim**

3587-S-AS-BI-2-6-D1 119150 113426 119150 113426 106418 106437 105219 103420

3593-S-RS-BI-4-5-D1 88310 83843 88310 83843 78987 79063 78563 78083

3594-S-FZ-BI-4-4-D1 101910 97309 101910 97309 91007 90997 90662 89718

3586-S-VF-BI-2-5-D1 124436 119216 124436 119216 113705 113690 113164 111634

Where

FWD_in: total number of the forward reads used in the analysis.

FWD_ctdpt: Total number of the forward reads passed the cutadapt primer trimming process

REV_in: total number of the reverse reads used in the analysis.

REV_ctdpt: Total number of the reverse reads passed the cutadapt primer trimming process.

denoisedF: Total number of the forward reads passed the DADA2 trimming and filtering process

denoisedR: Total number of the reverse reads passed the DADA2 trimming and filtering process

merged: : Total number of the successfully merged reads

nonchim: Total number of the merged and nonchimeric reads

During the DADA2 workflow 26731 unique amplicon sequence variants (ASV) were found in 48 samples. Out of

the 26731 ASVs the process idetified 7348 chimera sequences and 1001 ASVs that were shorter than the minimum

length (390 nt) or longer than the maximum length (500 nt).

**PERMANOVA analysis of the sample diversities between Groups**

**Table S7**: PERMANOVA analysis of the sample diversities between Groups D1 and D2 in Time sample feature – **warm continental climate**

|  | Df | Sum of Sqs | Mean Sqs | F | R² | *p* |
| --- | --- | --- | --- | --- | --- | --- |
| Time | 1 | 0.62 | 0.62 | 2.27 | 0.09 | 0.08 |
| Residuals | 21 | 5.76 | 0.27 | NA | 0.90 | NA |
| Total | 22 | 6.38 | NA | NA | 1.00 | NA |

**Table captions:** D1-first generation, D2 – second generation, Df – degree of freedom, Sum of Sqs - sum of squared distances divided by the number of plots, F - the ratio of variation between groups, R² - represents the proportion of variance in the data that is explained by the factors included in the model, p- statistic values (p ≤ 0.01 were considered as statistically significant differences, NA – statistic analyses not applicable.

**Table S8**: PERMANOVA analysis of the sample diversities between Groups D2 and D3 in Time sample feature - **warm continental climate**

|  | Df | Sum of Sqs | Mean Sqs | F | R² | *p* |
| --- | --- | --- | --- | --- | --- | --- |
| Time | 1 | 0.37 | 0.37 | 1.47 | 0.06 | 0.21 |
| Residuals | 22 | 5.52 | 0.25 | NA | 0.93 | NA |
| Total | 23 | 5.89 | NA | NA | 1.00 | NA |

**Table captions:** D2-second generation, D3 – third generation, Df – degree of freedom, Sum of Sqs - sum of squared distances divided by the number of plots, F - the ratio of variation between groups, R² - represents the proportion of variance in the data that is explained by the factors included in the model, p- statistic values (p ≤ 0.01 were considered as statistically significant differences, NA – statistic analyses not applicable.

**Table S9**: PERMANOVA analysis of the sample diversities between Groups D1 and D3 in Time sample feature - **warm continental climate**

|  | Df | Sum of Sqs | Mean Sqs | F | R² | *p* |
| --- | --- | --- | --- | --- | --- | --- |
| Time | 1 | 0.14 | 0.14 | 0.53 | 0.02 | 0.57 |
| Residuals | 21 | 5.79 | 0.27 | NA | 0.97 | NA |
| Total | 22 | 5.94 | NA | NA | 1.00 | NA |

**Table captions:** D1-first generation, D3 – third generation, Df – degree of freedom, Sum of Sqs - sum of squared distances divided by the number of plots, F - the ratio of variation between groups, R² - represents the proportion of variance in the data that is explained by the factors included in the model, p- statistic values (p ≤ 0.01 were considered as statistically significant differences, NA – statistic analyses not applicable.

**Table S10**: PERMANOVA analysis of the sample diversities between Groups TAS and TCZ in SoilType sample feature - **warm continental climate**

|  | Df | Sum of Sqs | Mean Sqs | F | R² | *p* |
| --- | --- | --- | --- | --- | --- | --- |
| SoilType | 1 | 0.44 | 0.44 | 1.94 | 0.11 | 0.12 |
| Residuals | 15 | 3.39 | 0.22 | NA | 0.88 | NA |
| Total | 16 | 3.83 | NA | NA | 1.00 | NA |

**Table captions:** TAS-Timis region, Aluviosol, TCZ – Timis region, Cernoziom, Df – degree of freedom, Sum of Sqs - sum of squared distances divided by the number of plots, F - the ratio of variation between groups, R² - represents the proportion of variance in the data that is explained by the factors included in the model, p- statistic values (p ≤ 0.01 were considered as statistically significant differences, NA – statistic analyses not applicable.

**Table S11**: PERMANOVA analysis of the sample diversities between Groups TAS and TPG in SoilType sample feature - **warm continental climate**

|  | Df | Sum of Sqs | Mean Sqs | F | R² | *p* |
| --- | --- | --- | --- | --- | --- | --- |
| SoilType | 1 | 0.77 | 0.77 | 3.77 | 0.20 | 0.06 |
| Residuals | 15 | 3.08 | 0.20 | NA | 0.79 | NA |
| Total | 16 | 3.85 | NA | NA | 1.00 | NA |

**Table captions:** TAS-Timis region, Aluviosol, TPG – Timis region Control garden soil, Df – degree of freedom, Sum of Sqs - sum of squared distances divided by the number of plots, F - the ratio of variation between groups, R² - represents the proportion of variance in the data that is explained by the factors included in the model, p- statistic values (p ≤ 0.01 were considered as statistically significant differences, NA – statistic analyses not applicable.

**Table S12**: PERMANOVA analysis of the sample diversities between Groups TAS and TPS in SoilType sample feature - **warm continental climate**

|  | Df | Sum of Sqs | Mean Sqs | F | R² | *p* |
| --- | --- | --- | --- | --- | --- | --- |
| SoilType | 1 | 0.37 | 0.37 | 1.46 | 0.08 | 0.19 |
| Residuals | 15 | 3.82 | 0.25 | NA | 0.91 | NA |
| Total | 16 | 4.19 | NA | NA | 1.00 | NA |

**Table captions:** TAS-Timis region, Aluviosol, TPS-Timis region, Preluvosol, Df – degree of freedom, Sum of Sqs - sum of squared distances divided by the number of plots, F - the ratio of variation between groups, R² - represents the proportion of variance in the data that is explained by the factors included in the model, p- statistic values (p ≤ 0.01 were considered as statistically significant differences, NA – statistic analyses not applicable.

**Table S13**: PERMANOVA analysis of the sample diversities between Groups TCZ and TPG in SoilType sample feature - **warm continental climate**

|  | Df | Sum of Sqs | Mean Sqs | F | R² | *p* |
| --- | --- | --- | --- | --- | --- | --- |
| SoilType | 1 | 0.12 | 0.12 | 0.45 | 0.02 | 0.67 |
| Residuals | 16 | 4.46 | 0.27 | NA | 0.97 | NA |
| Total | 17 | 4.59 | NA | NA | 1.00 | NA |

**Table captions:** TCZ-Timis region, Cernoziom, TPG-Timis region, Control garden soil, Df – degree of freedom, Sum of Sqs - sum of squared distances divided by the number of plots, F - the ratio of variation between groups, R² - represents the proportion of variance in the data that is explained by the factors included in the model, p- statistic values (p ≤ 0.01 were considered as statistically significant differences, NA – statistic analyses not applicable.

**Table** **S14**: PERMANOVA analysis of the sample diversities between Groups TCZ and TPS in SoilType sample feature - **warm continental climate**

|  | Df | Sum of Sqs | Mean Sqs | F | R² | *p* |
| --- | --- | --- | --- | --- | --- | --- |
| SoilType | 1 | 0.10 | 0.10 | 0.32 | 0.01 | 0.87 |
| Residuals | 16 | 5.20 | 0.32 | NA | 0.98 | NA |
| Total | 17 | 5.30 | NA | NA | 1.00 | NA |

**Table captions:** TCZ-Timis region Cernoziom, TPS-Timis region, Preluvosol, Df – degree of freedom, Sum of Sqs - sum of squared distances divided by the number of plots, F - the ratio of variation between groups, R² - represents the proportion of variance in the data that is explained by the factors included in the model, p- statistic values (p ≤ 0.01 were considered as statistically significant differences, NA – statistic analyses not applicable.

**Table S15**: PERMANOVA analysis of the sample diversities between Groups TPG and TPS in SoilType sample feature - **warm continental climate**

|  | Df | Sum of Sqs | Mean Sqs | F | R² | *p* |
| --- | --- | --- | --- | --- | --- | --- |
| SoilType | 1 | 0.24 | 0.24 | 0.80 | 0.04 | 0.42 |
| Residuals | 16 | 4.88 | 0.30 | NA | 0.95 | NA |
| Total | 17 | 5.13 | NA | NA | 1.00 | NA |

**Table captions:** TPG-Timis region, Control garden soil, TPS-Timis region, Preluvosol, Df – degree of freedom, Sum of Sqs - sum of squared distances divided by the number of plots, F - the ratio of variation between groups, R² - represents the proportion of variance in the data that is explained by the factors included in the model, p- statistic values (p ≤ 0.01 were considered as statistically significant differences, NA – statistic analyses not applicable.

**Table S16**: PERMANOVA analysis of the sample diversities between Groups D1 and D2 in Time sample feature - **temperate continental climate**

|  | Df | Sum of Sqs | Mean Sqs | F | R² | *p* |
| --- | --- | --- | --- | --- | --- | --- |
| Time | 1 | 0.14 | 0.14 | 0.35 | 0.01 | 0.98 |
| Residuals | 19 | 7.42 | 0.39 | NA | 0.98 | NA |
| Total | 20 | 7.56 | NA | NA | 1.00 | NA |

**Table captions:** D1-first generation, D2 – second generation, Df – degree of freedom, Sum of Sqs - sum of squared distances divided by the number of plots, F - the ratio of variation between groups, R² - represents the proportion of variance in the data that is explained by the factors included in the model, p- statistic values (p ≤ 0.01 were considered as statistically significant differences, NA – statistic analyses not applicable.

**Table 17**: PERMANOVA analysis of the sample diversities between Groups D2 and D3 in Time sample feature - **temperate continental climate**

|  | Df | Sum of Sqs | Mean Sqs | F | R² | *p* |
| --- | --- | --- | --- | --- | --- | --- |
| Time | 1 | 0.19 | 0.19 | 0.55 | 0.02 | 0.73 |
| Residuals | 19 | 6.59 | 0.34 | NA | 0.97 | NA |
| Total | 20 | 6.78 | NA | NA | 1.00 | NA |

**Table captions:** D2-second generation, D3 – third generation, Df – degree of freedom, Sum of Sqs - sum of squared distances divided by the number of plots, F - the ratio of variation between groups, R² - represents the proportion of variance in the data that is explained by the factors included in the model, p- statistic values (p ≤ 0.01 were considered as statistically significant differences, NA – statistic analyses not applicable.

**Table 18**: PERMANOVA analysis of the sample diversities between Groups D1 and D3 in Time sample feature - **temperate continental climate**

|  | Df | Sum of Sqs | Mean Sqs | F | R² | *p* |
| --- | --- | --- | --- | --- | --- | --- |
| Time | 1 | 0.31 | 0.31 | 0.88 | 0.04 | 0.43 |
| Residuals | 18 | 6.41 | 0.35 | NA | 0.95 | NA |
| Total | 19 | 6.73 | NA | NA | 1.00 | NA |

**Table captions:** D1-first generation, D3 – third generation, Df – degree of freedom, Sum of Sqs - sum of squared distances divided by the number of plots, F - the ratio of variation between groups, R² - represents the proportion of variance in the data that is explained by the factors included in the model, p- statistic values (p ≤ 0.01 were considered as statistically significant differences, NA – statistic analyses not applicable.

**Table 19**: PERMANOVA analysis of the sample diversities between Groups MAS and MEL in SoilType sample feature **temperate continental climate**

|  | Df | Sum of Sqs | Mean Sqs | F | R² | *p* |
| --- | --- | --- | --- | --- | --- | --- |
| SoilType | 1 | 0.54 | 0.54 | 1.35 | 0.09 | 0.20 |
| Residuals | 13 | 5.26 | 0.40 | NA | 0.90 | NA |
| Total | 14 | 5.81 | NA | NA | 1.00 | NA |

**Table captions:** MAS – Mures Region, Aluviosol entic, MEL – Preluvosol argic stagnic, Df – degree of freedom, Sum of Sqs - sum of squared distances divided by the number of plots, F - the ratio of variation between groups, R² - represents the proportion of variance in the data that is explained by the factors included in the model, p- statistic values (p ≤ 0.01 were considered as statistically significant differences, NA – statistic analyses not applicable.

**Table 20**: PERMANOVA analysis of the sample diversities between Groups MAS and MGS in SoilType sample feature **temperate continental climate**

|  | Df | Sum of Sqs | Mean Sqs | F | R² | *p* |
| --- | --- | --- | --- | --- | --- | --- |
| SoilType | 1 | 0.16 | 0.16 | 0.48 | 0.03 | 0.83 |
| Residuals | 13 | 4.35 | 0.33 | NA | 0.96 | NA |
| Total | 14 | 4.51 | NA | NA | 1.00 | NA |

**Table captions:** MAS – Mures Region, Aluviosol entic, MGS – Gleyosol, Df – degree of freedom, Sum of Sqs - sum of squared distances divided by the number of plots, F - the ratio of variation between groups, R² - represents the proportion of variance in the data that is explained by the factors included in the model, p- statistic values (p ≤ 0.01 were considered as statistically significant differences, NA – statistic analyses not applicable.

**Table 21**: PERMANOVA analysis of the sample diversities between Groups MAS and MVF in SoilType sample feature **temperate continental climate**

|  | Df | Sum of Sqs | Mean Sqs | F | R² | *p* |
| --- | --- | --- | --- | --- | --- | --- |
| SoilType | 1 | 0.15 | 0.15 | 0.46 | 0.03 | 0.87 |
| Residuals | 13 | 4.33 | 0.33 | NA | 0.96 | NA |
| Total | 14 | 4.48 | NA | NA | 1.00 | NA |

**Table captions:** MAS – Mures Region, Aluviosol entic, MVF – Control garden soil, Df – degree of freedom, Sum of Sqs - sum of squared distances divided by the number of plots, F - the ratio of variation between groups, R² - represents the proportion of variance in the data that is explained by the factors included in the model, p- statistic values (p ≤ 0.01 were considered as statistically significant differences, NA – statistic analyses not applicable.

**Table 22**: PERMANOVA analysis of the sample diversities between Groups MEL and MGS in SoilType sample feature **temperate continental climate**

|  | Df | Sum of Sqs | Mean Sqs | F | R² | *p* |
| --- | --- | --- | --- | --- | --- | --- |
| SoilType | 1 | 0.69 | 0.69 | 1.89 | 0.11 | 0.11 |
| Residuals | 14 | 5.12 | 0.36 | NA | 0.88 | NA |
| Total | 15 | 5.82 | NA | NA | 1.00 | NA |

**Table captions:** MEL – Preluvosol argic stagnic, MGS – Mures Region, Gleyosol, Df – degree of freedom, Sum of Sqs - sum of squared distances divided by the number of plots, F - the ratio of variation between groups, R² - represents the proportion of variance in the data that is explained by the factors included in the model, p- statistic values (p ≤ 0.01 were considered as statistically significant differences, NA – statistic analyses not applicable.

**Table 23**: PERMANOVA analysis of the sample diversities between Groups MEL and MVF in SoilType sample feature t**emperate continental climate**

|  | Df | Sum of Sqs | Mean Sqs | F | R² | *p* |
| --- | --- | --- | --- | --- | --- | --- |
| SoilType | 1 | 0.69 | 0.69 | 1.90 | 0.11 | 0.10 |
| Residuals | 14 | 5.09 | 0.36 | NA | 0.88 | NA |
| Total | 15 | 5.79 | NA | NA | 1.00 | NA |

**Table captions:** MEL – Preluvosol argic stagnic, MVF – Control garden soil, Df – degree of freedom, Sum of Sqs - sum of squared distances divided by the number of plots, F - the ratio of variation between groups, R² - represents the proportion of variance in the data that is explained by the factors included in the model, p- statistic values (p ≤ 0.01 were considered as statistically significant differences, NA – statistic analyses not applicable.

**Table 24**: PERMANOVA analysis of the sample diversities between Groups MGS and MVF in SoilType sample feature **temperate continental climate**

|  | Df | Sum of Sqs | Mean Sqs | F | R² | *p* |
| --- | --- | --- | --- | --- | --- | --- |
| SoilType | 1 | 0.12 | 0.12 | 0.42 | 0.02 | 0.77 |
| Residuals | 14 | 4.17 | 0.29 | NA | 0.97 | NA |
| Total | 15 | 4.30 | NA | NA | 1.00 | NA |

**Table captions:** MGS – Mures Region, Gleyosol, MVF – Control garden soil, Df – degree of freedom, Sum of Sqs - sum of squared distances divided by the number of plots, F - the ratio of variation between groups, R² - represents the proportion of variance in the data that is explained by the factors included in the model, p- statistic values (p ≤ 0.01 were considered as statistically significant differences, NA – statistic analyses not applicable.

**Table 25**: PERMANOVA analysis of the sample diversities between Groups D1 and D2 in Time sample feature - **cool cool continental climate**

|  | Df | Sum of Sqs | Mean Sqs | F | R² | *p* |
| --- | --- | --- | --- | --- | --- | --- |
| Time | 1 | 0.12 | 0.12 | 0.52 | 0.02 | 0.74 |
| Residuals | 22 | 5.14 | 0.23 | NA | 0.97 | NA |
| Total | 23 | 5.26 | NA | NA | 1.00 | NA |

**Table captions:** D1-first generation, D2 – second generation, Df – degree of freedom, Sum of Sqs - sum of squared distances divided by the number of plots, F - the ratio of variation between groups, R² - represents the proportion of variance in the data that is explained by the factors included in the model, p- statistic values (p ≤ 0.01 were considered as statistically significant differences, NA – statistic analyses not applicable.

**Table 26**: PERMANOVA analysis of the sample diversities between Groups D2 and D3 in Time sample feature - **cool cool continental climate**

|  | Df | Sum of Sqs | Mean Sqs | F | R² | *p* |
| --- | --- | --- | --- | --- | --- | --- |
| Time | 1 | 0.15 | 0.15 | 1.11 | 0.04 | 0.29 |
| Residuals | 22 | 3.12 | 0.14 | NA | 0.95 | NA |
| Total | 23 | 3.28 | NA | NA | 1.00 | NA |

**Table captions:** D2-second generation, D3 – third generation, Df – degree of freedom, Sum of Sqs - sum of squared distances divided by the number of plots, F - the ratio of variation between groups, R² - represents the proportion of variance in the data that is explained by the factors included in the model, p- statistic values (p ≤ 0.01 were considered as statistically significant differences, NA – statistic analyses not applicable.

**Table 27**: PERMANOVA analysis of the sample diversities between Groups D1 and D3 in Time sample feature - **cool cool continental climate**

|  | Df | Sum of Sqs | Mean Sqs | F | R² | *p* |
| --- | --- | --- | --- | --- | --- | --- |
| Time | 1 | 0.30 | 0.30 | 1.65 | 0.06 | 0.16 |
| Residuals | 22 | 4.02 | 0.18 | NA | 0.93 | NA |
| Total | 23 | 4.32 | NA | NA | 1.00 | NA |

**Table captions:** D1-first generation, D3 – third generation, Df – degree of freedom, Sum of Sqs - sum of squared distances divided by the number of plots, F - the ratio of variation between groups, R² - represents the proportion of variance in the data that is explained by the factors included in the model, p- statistic values (p ≤ 0.01 were considered as statistically significant differences, NA – statistic analyses not applicable.

**Table 28**: PERMANOVA analysis of the sample diversities between Groups SAS and SFZ in SoilType sample feature - **cool continental climate**

|  | Df | Sum of Sqs | Mean Sqs | F | R² | *p* |
| --- | --- | --- | --- | --- | --- | --- |
| SoilType | 1 | 0.10 | 0.10 | 0.42 | 0.02 | 0.93 |
| Residuals | 16 | 3.96 | 0.24 | NA | 0.97 | NA |
| Total | 17 | 4.06 | NA | NA | 1.00 | NA |

**Table captions:** SAS – Sepsi region Aluviosol coluvic, SFZ – Sepsi region Faeozion, Df – degree of freedom, Sum of Sqs - sum of squared distances divided by the number of plots, F - the ratio of variation between groups, R² - represents the proportion of variance in the data that is explained by the factors included in the model, p- statistic values (p ≤ 0.01 were considered as statistically significant differences, NA – statistic analyses not applicable.

**Table 29**: PERMANOVA analysis of the sample diversities between Groups SAS and SRS in SoilType sample feature - **cool continental climate**

|  | Df | Sum of Sqs | Mean Sqs | F | R² | *p* |
| --- | --- | --- | --- | --- | --- | --- |
| SoilType | 1 | 0.15 | 0.15 | 1.15 | 0.06 | 0.37 |
| Residuals | 16 | 2.09 | 0.13 | NA | 0.93 | NA |
| Total | 17 | 2.24 | NA | NA | 1.00 | NA |

**Table captions:** SAS – Sepsi region Aluviosol coluvic, SRS – Sepsi region Regosol calcaric, Df – degree of freedom, Sum of Sqs - sum of squared distances divided by the number of plots, F - the ratio of variation between groups, R² - represents the proportion of variance in the data that is explained by the factors included in the model, p- statistic values (p ≤ 0.01 were considered as statistically significant differences, NA – statistic analyses not applicable.

**Table 30**: PERMANOVA analysis of the sample diversities between Groups SAS and SVF in SoilType sample feature - **cool continental climate**

|  | Df | Sum of Sqs | Mean Sqs | F | R² | *p* |
| --- | --- | --- | --- | --- | --- | --- |
| SoilType | 1 | 0.18 | 0.18 | 0.86 | 0.05 | 0.47 |
| Residuals | 16 | 3.44 | 0.21 | NA | 0.94 | NA |
| Total | 17 | 3.62 | NA | NA | 1.00 | NA |

**Table captions:** SAS – Sepsi region, Aluviosol coluvic, SVF – Sepsi region Control garden soil, Df – degree of freedom, Sum of Sqs - sum of squared distances divided by the number of plots, F - the ratio of variation between groups, R² - represents the proportion of variance in the data that is explained by the factors included in the model, p- statistic values (p ≤ 0.01 were considered as statistically significant differences, NA – statistic analyses not applicable.

**Table 31**: PERMANOVA analysis of the sample diversities between Groups SFZ and SRS in SoilType sample feature - **cool continental climate**

|  | Df | Sum of Sqs | Mean Sqs | F | R² | *p* |
| --- | --- | --- | --- | --- | --- | --- |
| SoilType | 1 | 0.27 | 0.27 | 1.76 | 0.09 | 0.10 |
| Residuals | 16 | 2.52 | 0.15 | NA | 0.90 | NA |
| Total | 17 | 2.80 | NA | NA | 1.00 | NA |

**Table captions:** SFZ – Sepsi region Faeozion, SRS – Sepsi region Regosoil calcaric, Df – degree of freedom, Sum of Sqs - sum of squared distances divided by the number of plots, F - the ratio of variation between groups, R² - represents the proportion of variance in the data that is explained by the factors included in the model, p- statistic values (p ≤ 0.01 were considered as statistically significant differences, NA – statistic analyses not applicable.

**Table 32**: PERMANOVA analysis of the sample diversities between Groups SFZ and SVF in SoilType sample feature - **cool continental climate**

|  | Df | Sum of Sqs | Mean Sqs | F | R² | *p* |
| --- | --- | --- | --- | --- | --- | --- |
| SoilType | 1 | 0.11 | 0.11 | 0.46 | 0.02 | 0.78 |
| Residuals | 16 | 3.87 | 0.24 | NA | 0.97 | NA |
| Total | 17 | 3.98 | NA | NA | 1.00 | NA |

**Table captions:** SFZ – Sepsi region Faeozion, SVF – Sepsi region Control garden soil, Df – degree of freedom, Sum of Sqs - sum of squared distances divided by the number of plots, F - the ratio of variation between groups, R² - represents the proportion of variance in the data that is explained by the factors included in the model, p- statistic values (p ≤ 0.01 were considered as statistically significant differences, NA – statistic analyses not applicable.

**Table 33**: PERMANOVA analysis of the sample diversities between Groups SRS and SVF in SoilType sample feature - **cool continental climate**

|  | Df | Sum of Sqs | Mean Sqs | F | R² | *p* |
| --- | --- | --- | --- | --- | --- | --- |
| SoilType | 1 | 0.29 | 0.29 | 2.36 | 0.12 | 0.10 |
| Residuals | 16 | 2.00 | 0.12 | NA | 0.87 | NA |
| Total | 17 | 2.30 | NA | NA | 1.00 | NA |

**Table captions:** SRS – Sepsi region Regosol calcaric, SVF – Sepsi region Control garden soil, Df – degree of freedom, Sum of Sqs - sum of squared distances divided by the number of plots, F - the ratio of variation between groups, R² - represents the proportion of variance in the data that is explained by the factors included in the model, p- statistic values (p ≤ 0.01 were considered as statistically significant differences, NA – statistic analyses not applicable.
